# A Multi-Criteria Decision Support and Application to the Evaluation of the Fourth Wave of COVID-19 Pandemic

**DOI:** 10.3390/e24050642

**Published:** 2022-05-03

**Authors:** Constanta Zoie Radulescu, Marius Radulescu, Radu Boncea

**Affiliations:** 1 National Institute for Research and Development in Informatics, 8-10, Mareşal Averescu, 011455 Bucharest, Romania; zoie.radulescu@ici.ro (C.Z.R.); radu.boncea@ici.ro (R.B.); 2 “Gheorghe Mihoc-Caius Iacob” Institute of Mathematical Statistics and Applied Mathematics of the Romanian Academy, Calea 13 Septembrie, No. 13, 050711 Bucharest, Romania

**Keywords:** multi-criteria decision support, complex COVID-19 indicator, group AHP, extended entropy, COPRAS multi-criteria method, normalization, fourth COVID-19 wave

## Abstract

The COVID-19 pandemic caused important health and societal damage across the world in 2020–2022. Its study represents a tremendous challenge for the scientific community. The correct evaluation and analysis of the situation can lead to the elaboration of the most efficient strategies and policies to control and mitigate its propagation. The paper proposes a Multi-Criteria Decision Support (MCDS) based on the combination of three methods: the Group Analytic Hierarchy Process (GAHP), which is a subjective group weighting method; Extended Entropy Weighting Method (EEWM), which is an objective weighting method; and the COmplex PRoportional ASsessment (COPRAS), which is a multi-criteria method. The COPRAS uses the combined weights calculated by the GAHP and EEWM. The sum normalization (SN) is considered for COPRAS and EEWM. An extended entropy is proposed in EEWM. The MCDS is implemented for the development of a complex COVID-19 indicator called COVIND, which includes several countries’ COVID-19 indicators, over a fourth COVID-19 wave, for a group of European countries. Based on these indicators, a ranking of the countries is obtained. An analysis of the obtained rankings is realized by the variation of two parameters: a parameter that describes the combination of weights obtained with EEWM and GAHP and the parameter of extended entropy function. A correlation analysis between the new indicator and the general country indicators is performed. The MCDS provides policy makers with a decision support able to synthesize the available information on the fourth wave of the COVID-19 pandemic.

## 1. Introduction

The coronavirus SARS-CoV 2, the virus that generated COVID-19 disease, has been the main cause of important changes in people’s lives since it was identified at the end of 2019. The COVID-19 has received pandemic status in March 2020, as it spread to all continents and all countries in a relative short period of time. Sustained efforts are being made to limit the spread of the virus and to support and allow health systems to cope with the situation. The evolution of the pandemic generated by the SARS-CoV 2 virus differs from continent to continent, from country to country and from the virus variants. This situation posed many challenges to European healthcare systems. Most governments have implemented restrictive, sometimes intrusive decisions to reduce the spread of SARS-CoV 2.

The pandemic generated by the SARS-CoV 2 coronavirus is monitored, at the level of each country, every day, by several COVID-19 indicators.

Under these conditions, the correct evaluation and analysis of the situation caused by the COVID-19 pandemic can lead to the elaboration of the more efficient strategies at the country level. An important contribution in the elaboration of European policies is the understanding of the situation generated by the coronavirus infection (COVID-19) from several points of view. This may be achieved by calculating a complex indicator that takes into account several, separate, measured COVID-19 indicators. The problem of obtaining a complex COVID-19 indicator can be solved by a multi-criteria decision support based on multi-criteria method and/or weighting method or combination of methods.

New multi-criteria methods and combination of methods have been developed in the last decades. They were applied in various domains. Many of these methods are extensions or improvements of the existing methods [1,2,3].

The paper proposes a Multi-Criteria Decision Support (MCDS) based on the combination of three methods: GAHP [4,5], a subjective group weighting method, EEWM, an objective extended weighting method, and COPRAS [6,7], a multi-criteria method. The principal contribution of the paper is the MCDS. In MCDS, a generalization of Shannon entropy, which we shall call extended entropy, is proposed for computing objective weights. The method that uses this entropy is called EEWM. In addition, in MCDS, a linear combination between GAHP and EEWM weights is proposed. It includes both the uncertainty in the evaluation matrix and the contribution of the experience of a group of experts in the related field. The trade-off between the subjective involvement of experts and the objective evaluation in the final calculation of weights is controlled by a parameter.

The MCDS is implemented for the development of a new complex COVID-19 indicator called COVIND, that includes several countries COVID-19 indicators, over a fourth COVID-19 wave, for a group of European countries. Based on these indicators, a ranking of the countries is obtained. This indicator can identify which countries are more vulnerable to COVID-19 illnesses from several points of view (indicators) considered together.

The proposed MCDS may provide decision makers with a decision support capable of synthesizing the available information into a complex indicator. MCDS and COVIND can contribute to the correct evaluation and analysis of the pandemic situation and to the elaboration of strategies for mitigating the risks of spreading the infection by considering the multi-criteria aspect of the problem.

This paper is organized as follows. In the second section, a literature review on the application of multi-criteria methods in the study of the COVID-19 pandemic is presented. In the third section, the proposed decision support MCDS is described. Section 3.1 is dedicated to the selection of methods and Section 3.2 to MCDS input data description. In Section 3.3, MCDS stages were described. The GAHP weighting method in the context of the proposed decision support is presented in Section 3.4. The consistency check of the pairwise comparison matrices, the pairwise comparison matrices aggregation, and the calculation of the GAHP criteria weights by Dominant Eigenvalue (Power Method) is detailed. Section 3.5 presents the extended entropy function and the EEWM. In Section 3.6, the overall criteria weights calculation is detailed. Section 3.7 is dedicated to the COPRAS multi-criteria method. The normalized evaluation matrix is weighted. The COPRAS solutions and the ranks of the alternatives are calculated. In the fourth section, the MCDS is implemented for ranking and analysis of a set of 12 European countries for five COVID-19 indicators (criteria) in the fourth COVID-19 wave. An analysis is realized.

## 2. Literature Review

At present, there is a rich literature on the applications of multi-criteria methods to problems connected with COVID-19 in various domains such as health, economics, finance, and education. Some examples are: the grade assessment of COVID-19 Disease [8], estimating of the Brazilian health care system risk due to COVID-19 [9], evaluation of the available COVID-19 treatment options by MCDM techniques, fuzzy PROMETHEE and VIKOR [10], the selection of strategic guidelines for the healthcare system reorganization under the conditions of the COVID-19 pandemic, based on a multi-criteria methodology [11], evaluation and benchmarking the different diagnostic models for COVID19 based on multi-criteria decision-making [12], the initial spreading of COVID-19 in New York City in the conditions of the particulate matter PM2.5 air pollutant [13], a multi-factor weighted spatial analysis to understand how each country is impacted by the virus [14], quarantine decisions due to the COVID-19 pandemic [15], an integrated multi-criteria framework to evaluate the healthcare sector [16], and a SEIRD model (Susceptible, Exposed, Infective, Recovered and Deceased) for COVID-19 infection with a new parametrization that was proposed [17].

An analysis of the scientific literature reveals that the idea of using a complex indicator for the study of COVID-19 calculated by a multi-criteria method and a combination of weighting methods (EEWM and GAHP) is new.

There are several articles in which the indicators taken into account are different depending on the situation approached. No weighting methods are considered or equal weights are used for the criteria. Some examples are presented in the following.

The authors of [18] evaluated and analyzed the safety levels of 100 regions in the world in terms of COVID-19 using TOPSIS, VIKOR and COPRAS methods. The main criteria of quarantine efficiency, government efficiency of risk management, monitoring and detection, health readiness, regional resilience, and emergency preparedness are used in the evaluation of countries and regions. No weighting methods are used or a hybrid combination of methods is used.

In [19], the multi-criteria method TOPSIS is used to generate a ranking of countries based on health, society and work criteria that define three areas severely affected by the COVID-19. TOPSIS calculates a score that cumulates the considered criteria. No weighting methods are used in the paper.

In the study by [20], MCDM techniques such as Weighted Sum Model (WSM), Weighted Product Model (WPM), Weighted Aggregated Sum Product Assessment (WASPAS), and TOPSIS were used, and regions were listed according to control measures implemented to stop the spread of COVID-19. No weighting methods are used.

In [21], the authors computed an index of vulnerability at the state and district levels of India based on 15 indicators across the following five domains: socioeconomic, demographic, housing and hygiene, epidemiological, and health system. A percentile ranking method which is a simple method to compute both domain-specific and overall vulnerability is used. The equal weights were assigned to each indicator for calculating the domain vulnerability.

The aim of the paper [22] is to generate an area-level COVID-19 Pandemic Vulnerability Index (CPVI) and to assess its correlation with COVID-19 cases. Using factor analysis, four latent factors were identified and named as sociodemographic, medical conditions, transportation, and land use. No weighting methods are used in the paper.

Paper [23] presents a comparison of the OECD countries’ performance during the COVID-19 pandemic with the Global Health Security Index. No weighting methods were used for the criteria weights. The cumulative score involving all four variables of interest was equally weighted for each criterion. This was achieved by calculating the average rank for the cumulative score.

In [24], several factors that vary across geographies and can determine adverse effects on the National Health System during a pandemic, such as age, existing disease prevalence, medical resource availability, and deprivation are combined in order to obtain an indicator of community-level vulnerability that shows which areas are more exposed. The new indicator may help policy makers to mitigate the impact of similar pandemics. No weighting methods are used in the paper.

In [25], an indicator was developed, comprising appropriate components for assessing the level of wealth and “happiness” of all the Romanian counties. This indicator was used for approaching the entrepreneurial resilience during times of crisis caused by the COVID-19 pandemic in Romania in multiple manners according to its diverse forms. No weighting methods were used for the criteria. The indicator is calculated by summing the normalized indicators.

In [26], the correlation between tourism vulnerability and the COVID-19 pandemic was investigated. The authors proposed a vulnerability indicator for the tourism in Spain provinces. The multi-criteria method used is DEA, and the weighting method used is Principal Component Analysis.

For the control and diagnosis of COVID-19 in the paper [27], a spherical intelligent fuzzy decision model is proposed. This model is based on TOPSIS and COPRAS methods under a spherical fuzzy environment.

A comprehensive review and an analysis of the multidisciplinary fields, carried out by different MCDM concerning COVID-19 in complex case studies, are provided in the paper [28].

In the paper [29], for estimating and choosing the best alternative of health care waste treatment, the COPRAS method in a Pythagorean fuzzy set (PFS) is used. A new entropy measure on PFSs is proposed, and its validity is studied.

## 3. The Multi-Criteria Decision Support—MCDS

### 3.1. Selection of Methods

The MCDS is based on the combination of GAHP, EEWM and COPRAS methods. The overall criteria weights are calculated as a linear combination of criteria weights obtained with the GAHP and EEWM. These weights are used in COPRAS.

The weight associated to each criterion reflects the relative importance of the criterion. The determination of the weights can be performed by an expert (evaluator, decision-maker) or by a group of experts (evaluators, decision-makers). A critical input to most multi-criteria methods is the assignment of criteria weights. These can be obtained by subjective, objective or a combination of weighting methods [30].

The determination of the subjective weights is based on the opinion and experience of the experts. In order to obtain judgments on the criteria, experts answer a set of questions about the criteria. Determining subjective weights is often time consuming and depends on the expertise of the specialists involved.

Subjective weighting methods are based on the experts’ preferences, while the emphasis of the objective methods is put on the statistical evaluation of data taken from the evaluation matrix. In cases when experts’ opinions are difficult to obtain, one can use objective weights [31]. Each of these weighting methods has its advantages and disadvantages.

The lack of experience, imprecise information, the limited capability of the expert for analyzing and correlating criteria may have a negative impact on the precision of the weights assigned to criteria. A disadvantage of the objective methods is that they do not benefit from the expert’s experience.

None of the two weightings methods, subjective and objective, are perfect, and the integrated weighting method might be the most appropriate for determining the criteria weights. A combination of a subjective weighting method and an objective weighting method would ensure the combination of the experience of experts who know the field well with the measurement of the existing uncertainty in the evaluation matrix. The combination is controlled by a parameter that ensures the level of subjectivity (objectivity) in the calculation of the weights of the criteria.

Examples of objective weighting methods are the Entropy method [32,33,34], CRITIC (Criteria Importance Through Intercriteria Correlation) [35], and the Weighted Least-Square Method [36]. Example of subjective weighting methods are the Simple Multi-Attribute Rating Technique (SMART) [37], Analytical Hierarchy Process (AHP) [38,39], Step-Wise Weight Assessment Ratio Analysis (SWARA) [40], Weighted Aggregated Sum Product Assessment (WASPAS) [41], and Best–Worst Method (BWM) [42]. A summary of criteria weighting methods is presented in [43].

One of the effective and appropriate subjective weighting methods for group decision making is GAHP. A group approach was chosen because the diversity of stakeholders can have direct effects on the quality of results as well as the decision-making procedure. The GAHP is a structured method for organizing and analyzing complex decisions. Group AHP is suitable when the number of criteria is not very large. The method was chosen because it has a sound mathematical foundation, has a Saaty evaluation scale for the evaluation in pairs of criteria, is systematic, concise and calculates a consistency ratio that ensures a correct comparison in pairs, without inconsistencies.

The concept of information entropy was first introduced by Shannon [32]. Shannon and Weaver’s [33] entropy is a measure of uncertainty in information theory that indicates the inherent contrast intensity of the corresponding criteria. It can measure the amount of useful information in the evaluation matrix. When the difference between criteria values is high, then the entropy is small. When a criterion provides more useful information, the value of the corresponding weight should be higher [44].

One of the popular objective weights methods for obtaining criteria weights is the entropy weighting method (EWM). The entropy and EWM method were used in various other problems. In [45], EWM was used in the selection of an industrial robot for the arc welding operation. The TOPSIS method, combined with the EWM, was used to improve freight selection decisions [46]. In [47], EWM was used for the selection of Femoral Component material for total knee replacement. The paper [48] introduced the EWM to develop a combination prediction model for the ionospheric F2 layer critical frequency. The weights for calculating a multivariate index of sustainable development are calculated with the EWM in [49]. A gray EWM and a gray relation–projection pursuit model was proposed in [50] to calculate the weights of criteria for soybean irrigation schedule in the Huaibei Plain. In [51], EWM was used for providing collective information of the entropy weights methodology applied to different machining operations which compute the objective weights. A bibliometric analysis of EWM for multi-objective optimization in machining operations was made in [52]. The EWM is widely used compared to other objective weighting methods.

The EEWM, proposed in this paper, uses an extended definition of entropy that is a generalization of Shannon entropy. There are several generalizations of Shannon entropy; see for example [53]. The most important generalized entropies are Rényi entropy [54], Tsallis entropy [55], Havrda and Charvát entropy [56], and Kapur entropy [57]. The generalized entropies have played an important role in thermodynamic, statistical and informational systems.

Let $\Delta_{m}=\left\{\left(p_{1},p_{2},\ldots ,p_{m}\right)\in {\mathbb{R}}^{m}:\overset{m}{\underset{k=1}{\sum }}p_{k}=1\;\mathrm{and}\;p_{i}>0,\;i=1,2,\ldots ,m\right\}$ be the standard simplex. Examples of entropy functions are:

Shannon entropy [32]:

(1) $$f_{1}\left(p_{1},p_{2},\ldots ,p_{m}\right)=-\sum_{k=1}^{m}p_{k}lnp_{k};\left(p_{1},p_{2},\ldots ,p_{m}\right)\in \Delta_{m}$$

Renyi entropy [54]:

(2) $$f_{2}\left(p_{1},p_{2},\ldots ,p_{m}\right)=\frac{1}{1-q}ln\overset{m}{\underset{k=1}{\sum }}p_{k}^{q};\;\left(p_{1},p_{2},\ldots ,p_{m}\right)\in \Delta_{m}$$

Here, q≠1, q>0. One can easily check that $f_{2}$ is concave if 0 < *q* < 1. Renyi entropy reduces to Shannon entropy as a limiting case when *q* → 1.

Tsallis entropy [55]:

(3) $$f_{3}\left(p_{1},p_{2},\ldots ,p_{m}\right)=\frac{1}{1-q}\left(\sum_{k=1}^{m}p_{k}^{q}-1\right);\left(p_{1},p_{2},\ldots ,p_{m}\right)\in \Delta_{m}$$

Here, q≠1, q≥0. Tsallis entropy reduces to Shannon entropy as a limiting case when *q* → 1.

Kapur entropy [57]:

(4) $$f_{4}\left(p_{1},p_{2},\ldots ,p_{m}\right)=\frac{1}{1-q}ln\left(\frac{\sum_{k=1}^{m}p_{k}^{q+\beta -1}}{\sum_{k=1}^{m}p_{k}^{\beta }}\right);\;\left(p_{1},p_{2},\ldots ,p_{m}\right)\in \Delta_{m}$$

Here, q≠1, q>0, β>0, q+β−1>0. The above function reduces to Renyi entropy when *β* = 1. Further, it reduces to Shannon entropy when *β* = 1 and *q* → 1.

In our study, we shall propose a generalization of Shannon entropy, which we shall call extended entropy.

(5) $$f_{5}\left(p_{1},p_{2},\ldots ,p_{m}\right)=-\overset{m}{\underset{k=1}{\sum }}p_{k}^{a}ln(p_{k});\;\left(p_{1},p_{2},\ldots ,p_{m}\right)\in \Delta_{m}$$

Note that $\;f_{5}$ is concave for a∈[0.5;1]. The Shannon entropy is obtained for *a* = 1.

We shall use the extended entropy in the classical entropy weighting method for finding objective weights. We shall call EEWM the resulting entropy weights method.

A linear combination between GAHP and EEWM weights is proposed. The trade-off between the subjective involvement of experts and the objective evaluation in the final calculation of weights is controlled by the parameter μ∈[0,1].

The advantages of using the parameter μ that controls the trade-off between the objective and the subjective weighting is that the decision maker can face various real-life situations. For example,

1. If there are no experts to help in the evaluations, then the parameter will be chosen to be equal to zero.
2. If there are some experts for the evaluations in which there is a little confidence, one can take the values of the parameter close to zero.
3. If there are several experts for the evaluations in which there is great confidence, one can take the values of the parameter close to one or even equal to one.

In MCDS, the COPRAS multi-criteria method is selected.

Examples of multi-attribute methods frequently used in the practice are: SAW [34,58], TOPSIS [34], VIKOR [59,60], COPRAS [6,7], AHP [38,39], PROMETHEE [61] and ELECTRE III [62].

The COmplex PRoportional ASsessment (COPRAS) method was introduced by Zavadskas, Kaklauskas, and Sarka in 1994 [6,7]. In this method, the ranking of alternatives is performed using the value evaluation of maximizing and minimizing indexes. A comparative analysis of SAW and COPRAS was realized in [63]. A recent comparative analysis of TOPSIS, VIKOR and COPRAS was performed in [18]. The paper [64] presents a state-of-the-art literature survey on COPRAS applications and methodologies.

The COPRAS method is simple, logical, easy to understand for non-specialists in the field and provides a rational basis for decision making. Although it is a relatively new method, it has proven its effectiveness. However, the COPRAS method involves establishing weights associated with the selected criteria. It is based on an evaluation matrix (criteria alternatives) with quantitative values. In the COVID problem, the arrays of the evaluation matrix have numerical, concrete values given by the COVID-19 indicators for the fourth wave.

The choice of the methods VIKOR and ELECTRE III involves the user’s subjective contribution in setting parameters (the selection of threshold parameters). The subjective involvement of experts was preferred only in the case of calculating the criteria weights.

The choice of the COPRAS method was based on the advantages of this method compared to other multi-attribute methods for the studied problem. One of these advantages is that EEWM and COPRAS use the same normalization method (sum normalization, SN) for the evaluation matrix. The use of different normalization methods, when a combination of methods is applied, may lead to disruption of the results.

### 3.2. MCDS Input Data

The input data in the GAHP method are:

- $E=\left\{E_{1},\;E_{2},\ldots ,\;E_{p}\right\}$ the set of p≥1  experts.
- *C* is the set of *n* criteria $C=\left\{C_{1},\;C_{2},\ldots ,\;C_{n}\right\}$. A criterion *C_i_* from the set *C* is measured using a measure unit. They are two types of criteria: min criteria (those for which the decreasing values are better) and max criteria (those for which the increasing values are better). A weight can be calculated for each criterion.
- α is the threshold for acceptance of inconsistency. In our study, we shall take α = 0.1.

The input data in the EEWM are:

- $A=\left\{A_{1},\;A_{2},\ldots ,\;A_{m}\right\}$ the set of *m* alternatives.
- *C* is the set of *n* criteria (same as in GAHP).
- *Q* is the evaluation matrix alternatives criteria.
- Parameter a∈[0.5;1] of the extended entropy function.
- The input data in the overall criteria weights are:
- Parameter μ ∈ [0; 1] that shows the trade-off between subjective and objective weights.
- GAHP weights and EEWM weights.

The input data in the COPRAS are:

- $A=\left\{A_{1},\;A_{2},\ldots ,\;A_{m}\right\}$ is the set of *m* alternatives (same as in EEWM).
- *C* is the set of *n* criteria (same as in GAHP and EEWM).
- $\bar{Q}$ is the normalized evaluation matrix alternatives criteria (same as in EEWM).
- Overall criteria weights.

### 3.3. MCDS Stages

**Figure 1:**
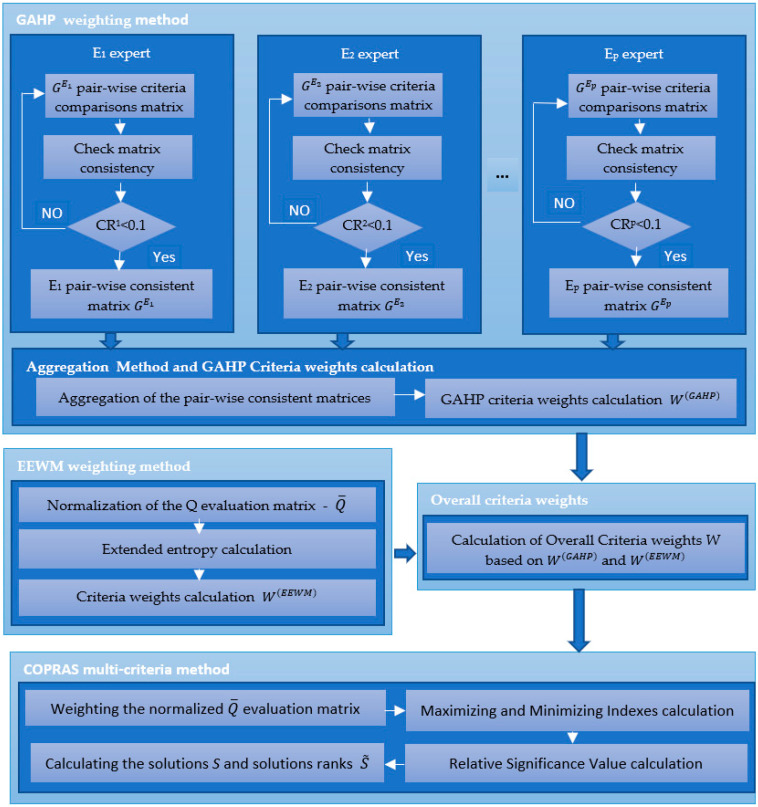
The proposed MCDS approach.

MCDS has the following stages (Figure 1):

1. Construction of input data in the MCDS;
2. Application of the GAHP weighting method:
  a. Checking the consistency and aggregation of the pairwise comparison matrices $G^{E_{r}}$;
  b. Calculation of the GAHP criteria weights $W^{\left(GAHP\right)}$;
3. Application of the EEWM and overall criteria weights:
  c. Normalized evaluation matrix calculation;
  d. Extended entropy calculation;
  e. Calculation of EEWM criteria $W^{\left(EEWM\right)}$;
  f. Overall criteria weights calculation $W^{\left(T\right)}$;
4. Application of the COPRAS method:
  g. Weighted normalized evaluation matrix calculation;
  h. Maximizing indexes and minimizing indexes calculation;
  i. Relative significance value calculation;
  j. Calculation of COPRAS solutions S and COPRAS solutions ranks $\tilde{S}$.

The details of the MCDS steps are presented in the following sections.

### 3.4. Group Analytic Hierarchy Process Weighting Method

Each expert from the set *E* of p experts makes sets of pairwise comparisons between criteria from the criteria set *C* based on Saaty’s 1–9 scale. For each expert *E_r_* from the set *E* of experts, a *n* × *n* pairwise comparison matrix $G^{E_{r}}=\left(g_{ij}^{\left(E_{r}\right)}\right)$ is obtained. We have $g_{ii}^{\left(E_{r}\right)}=1;\;g_{ij}^{\left(E_{r}\right)}=g_{ji}^{\left(E_{r}\right)};\;i,j=1,2,\ldots ,n;\;r=1,2,\ldots ,p.$ The element $g_{ij}^{\left(E_{r}\right)}$ denotes the evaluation of pairwise comparison of the $E_{r}$-th expert on the degree the criterion *C_i_*, which influences the criterion *C_j_* on the Saaty scale (Table 1).

Each matrix $G^{\left(E_{r}\right)}$ is verified for consistency. For each matrix $G^{\left(E_{r}\right)},$ the maximum eigenvalue $\lambda_{\max}^{\left(E_{r}\right)}$ and consistency index $CI^{\left(E_{r}\right)}$ are determined:

(6) $$CI^{\left(E_{r}\right)}=\frac{\lambda_{\max}^{(E_{r)}}-n}{n-1}$$

The consistency ratio $CR^{\left(E_{r}\right)}$ for each matrix $G^{\left(E_{r}\right)}$ is calculated:

(7) $$CR^{\left(E_{r}\right)}=\frac{CI^{\left(E_{r}\right)}}{RI}$$

where *RI* is the random consistency index obtained from Table 2.

If $CR^{\left(E_{r}\right)}\leq \alpha$, then the matrix $G^{\left(E_{r}\right)}$ is consistent.

If $CR^{\left(E_{r}\right)}>\alpha$, then the expert $E_{r}$ must correct his pairwise comparison.

The matrices $G^{E_{r}}$ are aggregated. There are several methods to obtain a group aggregation [65]. The comparison matrix in pairs $G=\left(g_{ij}\right)$ of the group of experts *E* will be calculated as follows:

(8) $$g_{ij}=\overset{p}{\underset{r=1}{\prod }}{\left(g_{ij}^{\left(E_{r}\right)}\right)}^{1/p},\;i,j=1,2,\ldots ,\;n$$

The GAHP criteria weights are calculated in the proposed MCDS using the Dominant Eigenvalue (Power Method).

The Dominant Eigenvalue algorithm used in MCDS is presented in the following:

**Step 1.** The initial elements are defined:

The initial eigenvector is defined: $V^{\left(0\right)}=\left(v_{i}^{\left(0\right)}\right)$ where $v_{i}^{\left(0\right)}=1;\;i=1,2,\ldots ,n$.

The error tolerance ε=0.000000001 and calculated error *ERR* = 10 is fixed.

The initial scaled vector ${\bar{V}}^{\left(0\right)}$ is defined: ${\bar{V}}^{\left(0\right)}=\left({\bar{v}}_{i}^{\left(0\right)}\right)$ where ${\bar{v}}_{i}^{\left(0\right)}=1;\;i=1,2,\ldots ,n$.

The initial normalized vector ${\overset{=}{V}}^{\left(0\right)}$ is defined: ${\overset{=}{V}}^{\left(0\right)}=\left({\overset{=}{v}}_{i}^{\left(0\right)}\right)$

$${\overset{=}{v}}_{i}^{\left(0\right)}=1/\overset{n}{\underset{j=1}{\sum }}{\bar{v}}_{j}^{\left(0\right)};\;i=1,2,\ldots ,n$$

The initial iteration number *k* = 1.

**Step 2.** The greatest eigenvalue and the corresponding eigenvector are calculated iteratively:

While ERR>ε then:

$V^{\left(k\right)}=\left(v_{i}^{\left(k\right)}\right)$ where $v_{i}^{\left(k\right)}=\overset{n}{\underset{j=1}{\sum }}g_{ij}*{\bar{v}}_{i}^{\left(k-1\right)}$ is computed.

The vector $V^{\left(k\right)}$ is scaled: ${\bar{V}}^{\left(k\right)}=\left({\bar{v}}_{i}^{\left(k\right)}\right);\;b_{k}=\underset{1\leq i\leq n}{\max}v_{i}^{\left(k\right)}$; ${\bar{v}}_{i}^{\left(k\right)}=\left(\frac{v_{i}^{\left(k\right)}}{b_{k}}\right)$.

The vector ${\bar{V}}^{\left(k\right)}$ is normalized: ${\overset{=}{V}}^{\left(k\right)}=\left({\overset{=}{v}}_{i}^{\left(k\right)}\right)$ where ${\overset{=}{v}}_{i}^{\left(k\right)}=\left(\frac{{\bar{v}}_{i}^{\left(k\right)}}{\sum_{j=1}^{n}{\bar{v}}_{j}^{\left(k\right)}}\right)$.

The approximative eigenvalue at iteration *k*, $EIG^{\left(k\right)}=\overset{n}{\underset{i=1}{\sum }}\left(\left(\overset{n}{\underset{j=1}{\sum }}g_{ij}\right)*{\overset{=}{v}}_{i}^{\left(k\right)}\right)$ is computed.

The error at iteration *k*, $ERR_{k}=\overset{n}{\underset{i=1}{\sum }}{\left({\overset{=}{v}}_{i}^{\left(k-1\right)}-{\overset{=}{v}}_{i}^{\left(k\right)}\right)}^{2}$ is calculated.

*k* = *k* + 1.

End While

If $ERR_{k}\leq \;\varepsilon$, then the approximation of the greatest eigenvalue is $EIG^{\left(k-1\right)}$ and the corresponding eigenvector (whose entries are the weights) is ${\overset{=}{V}}^{\left(k\right)}$.

**Step 3.** $W^{\left(GAHP\right)}={\overset{=}{V}}^{\left(k\right)}$ the GAHP criteria weights where:

$$W^{\left(GAHP\right)}=\left(w_{j}^{\left(GAHP\right)}\right);\;j=1,2,\ldots ,n.$$

The weights have numerical values in the range (0,1) and $\overset{n}{\underset{j=1}{\sum }}w_{j}^{\left(GAHP\right)}=1$.

### 3.5. Extended Entropy Weighting Method—EEWM

In order to have a valid comparison, the matrix *Q* must be normalized. Normalization methods usually map criteria with different measurement units to a common scale in the interval [0; 1]. The matrix *Q* is normalized with sum normalization (SN).

The entries of normalized matrix $\bar{Q}=({\bar{q}}_{ij})$ are calculated as follows:

(9) $${\bar{q}}_{ij}=\frac{q_{ij}}{\sum_{k=1}^{m}q_{kj}},\;i=1,2,\ldots ,m;\;j=1,2,\ldots ,n$$

A value in the range [0.5; 1] for the parameter *a* is selected. Then, $e_{j}$ is calculated with the normalized extended entropy:

(10) $$e_{j}=-\frac{m^{a-1}}{ln\left(m\right)}\;\sum_{i=1}^{m}{\bar{q}}_{ij}^{a}ln({\bar{q}}_{ij}),\;0\leq e_{j}\leq 1;\;j=1,2,\ldots ,n,$$

Recall that for *a* = 1, the Shannon entropy is obtained.

The arrays of the vector of criteria weights $W^{\left(EEWM\right)}=\left(w_{j}^{\left(EEWM\right)}\right)$ based on the entropy concept are calculated:

(11) $$w_{j}^{\left(EEWM\right)}=\frac{1-e_{j}}{\sum_{k=1}^{n}\left(1-e_{k}\right)},\;j=1,2,\ldots ,n$$

The weights have numerical values in the range (0; 1) and $\overset{n}{\underset{j=1}{\sum }}w_{j}^{\left(EEWM\right)}=1$.

### 3.6. Overall Criteria Weights Calculation

The set of overall criteria weights $W=\left(w_{j}\right)$ is obtained by a linear combination of GAHP criteria weights $W^{\left(GAHP\right)}=\left(w_{j}^{\left(GAHP\right)}\right)$ and entropy criteria weights $W^{\left(EEWM\right)}=\left(w_{j}^{\left(EEWM\right)}\right)$:

(12) $$w_{j}=\mu *w_{j}^{\left(GAHP\right)}+\left(1-\mu \right)*w_{j}^{\left(EEWM\right)},\;j=1,2,\ldots ,n$$

The trade-off between the subjective involvement of experts and the objective evaluation in the final calculation of weights is controlled by a parameter μ ∈ [0; 1]. The value of the parameters can be set depending on the degree of trust in the experts’ expertise.

In the case where there are no evaluators or there is little confidence in the evaluators’ expertise, the choice of μ is 0 or close to 0. In the case that there is great confidence in the evaluators’ expertise, the choice of μ is close to 1.

### 3.7. COPRAS Method

In the following, COPRAS solutions, and alternatives ranks are calculated.

The weighted normalized matrix $\overset{=}{Q}=({\overset{=}{q}}_{ij})$ is calculated based on the normalized matrix $\bar{Q}=({\bar{q}}_{ij})$, (cf. Equation (9)) and the weights vector $W=\left(w_{j}\right)$ (cf. Equation (12)) as follows:

(13) $${\overset{=}{q}}_{ij}=w_{j}*\;{\bar{q}}_{ij}$$

Let $M^{1}=\left\{j\in \left\{1,2,\ldots ,n\right\}:C_{j}\;\mathrm{is}\;a\max\mathrm{criterion}\right\}.$

Let $M^{2}=\left\{j\in \left\{1,2,\ldots ,n\right\}:C_{j}\;\mathrm{is}\;a\min\mathrm{criterion}\right\}.$

The Maximizing Indexes (for max criteria set $M^{1}$) $A^{+}=\left(a_{i}^{+}\right)$ and Minimizing Indexes (for min criteria set $M^{2}$) $A^{-}=\left(a_{i}^{-}\right),\;i=1,2,\ldots ,m$ are calculated as:

(14) $$a_{i}^{+}=\underset{j\in M^{1}}{\sum }{\overset{=}{q}}_{ij}\;,$$

(15) $$a_{i}^{-}=\underset{j\in M^{2}}{\sum }{\overset{=}{q}}_{ij}$$

For each i=1,2,…,m, are calculated the Relative Significance Value, the vector $P=\left(p_{i}\right)$ and the COPRAS solution $S=\left(s_{i}\right)$:

(16) $$p_{i}=a_{i}^{+}+\frac{\sum_{k=1}^{m}a_{k}^{-}}{a_{i}^{-}*\sum_{k=1}^{m}\left(\frac{1}{a_{k}^{-}}\right)};\;i=1,2,\ldots ,m$$

(17) $$s_{i}=p_{i}/\underset{k}{\max}p_{k}$$

The best alternative is $A_{i}$ for which $s_{i}=\underset{k}{\max}s_{k}$.

The vector of solution ranks is $\tilde{S}=\left({\tilde{s}}_{i}\right).$

## 4. Case Study

In the following, we shall apply the above-described MCDS to rank a group of European countries, in the context of the fourth wave of the SARS-CoV 2 pandemic based on COVIND indicators. The group of countries for which the analysis is conducted is considered as the set of alternatives. A set of COVID-19 indicators for the fourth wave is considered as the set of criteria. The COPRAS solutions are considered countries’ COVIND indicators.

The criteria (set *C*) in MCDS are:

- The slope of the fourth COVID wave in 2021 (*C*_1_). Here, by the slope, we understand the ratio between the number of new cases, in the period of time this number (we shall consider the smooth number) is increasing, and the number of days in the above-mentioned period. One can easily see that a great slope is not desirable, since the hospitals have a limited capacity and cannot treat more patients than they can handle;
- New cases smoothed/1 million inhabitants (*C*_2_);
- New deaths smoothed/1 million inhabitants (*C*_3_);
- Patients in intensive care units/1 million inhabitants (*C*_4_);
- New tests smoothed/1 million inhabitants (*C*_5_).

These criteria can be compared because they are calculated in relation to the population of a country (per 1 million inhabitants).

There are two types of indicators: min indicators: *C*_1_, *C*_2_*, C*_3_*, C*_4_ and max indicators: *C*_5_.

The set of European countries was selected according to several conditions:

- European countries with a population of more than 5 million;
- Countries for which data were available for the all selected criteria;
- Countries where there was an obvious COVID-19 fourth wave.

Important sources of information for MCDS were:

1. COVID-19 Data Repository of the Center for Systems Science and Engineering (CSSE) at Johns Hopkins University [66]. Source data for COVID-19 are daily updated from 22 January 2020. The data come from governments, national and sub-national agencies around the world. Here are links to aggregate data sources for COVID-19. Two of them are the World Health Organization (WHO) [67] and European Centre for Disease Prevention and Control (ECDC) [68].
2. Worldometer [69]. For the COVID-19 data, Worldometer collects data from official reports, directly from the government’s communication channels or indirectly, through local media sources when deemed reliable (5000 sources).
3. The COVID-19 dataset, a collection of the COVID-19 data, maintained by The Oxford Martin School and University of Oxford. It is updated daily throughout the duration of the COVID-19 pandemic [70].

From the 48 European countries for which COVID-19 data are available, 25 European countries with more than 5 million inhabitants [69] were considered. These countries are: Austria, Belarus, Belgium, Bulgaria, Czechia, Denmark, Finland, France, Germany, Greece, Hungary, Italy, Netherlands, Norway, Poland, Portugal, Romania, Russia, Serbia, Slovakia, Spain, Sweden, Switzerland, Ukraine, and the United Kingdom. Countries with no data to all criteria are: Belarus, Greece, Hungary, Norway, Poland, Russia, and Ukraine. Belarus and Poland have no data [70] for: patients in intensive care units/1 million inhabitants (C4), new tests smoothed/1 million inhabitants (C5). Greece, Hungary, Norway, Russia, and Ukraine have no data on: patients in intensive care units/1 million inhabitants (C4) [63]. From the remaining European countries were selected countries where there was an obvious COVID-19 fourth wave in the period considered.

We preferred to choose a smaller number of countries to make it easier to highlight the proposed application of MCDS. Decision makers can apply the proposed MCDS for any set of countries for which an analysis is wanted and for any pandemic wave.

In MCDS, the European countries selected (the set *A*) were: Austria (*A*_1_), Belgium (*A*_2_), Bulgaria (*A*_3_), Czechia (*A*_4_), France (*A*_5_), Germany (*A*_6_), Italy (*A*_7_), Romania (*A*_8_), Slovakia (*A*_9_), Spain (*A*_10_) Switzerland (*A*_11_) and Serbia (*A*_12_).

The set *A*_1_, *A*_2_, *A*_5_, *A*_6_, *A*_7_, *A*_10_ and *A*_11_ is a group of Western European countries.

The set *A*_3_, *A*_4_, *A*_8_, *A*_9_ and *A*_12_ is a group of Eastern European countries.

Each country from the set *A* was monitored by COVID-19 indicators in the fourth wave in 2021.

The entries of the *Q* matrix are calculated taking into account existing information on the Internet.

**Figure 2:**
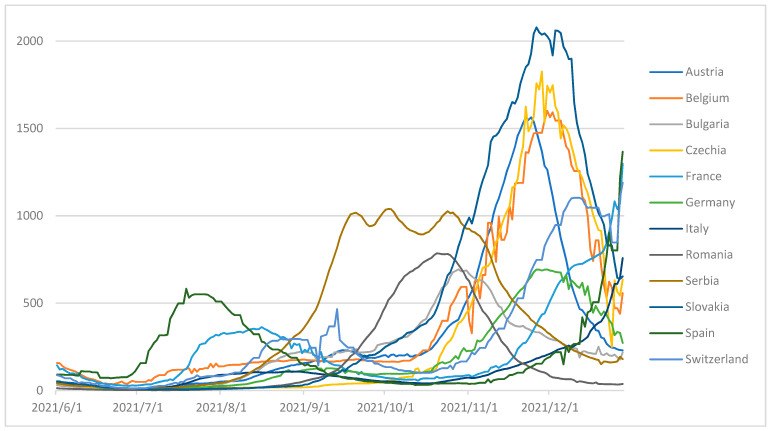
Number of smoothed daily new confirmed COVID-19 cases/1 million people, for the fourth wave, for the selected countries.

The fourth wave of COVID-19 happened differently in the European countries selected in the MCDS. In some countries, it started earlier than in others. The period is different; the slope corresponding to the period the number of new cases was increasing, was different and the peak of the wave was higher or lower. A situation of the number of smoothed daily new confirmed COVID-19 cases/1 million people, for the fourth wave, for the selected countries, is illustrated in Figure 2. The entire period considered of fourth wave COVID-19, for these countries, is between 1 June 2021 and 28 December 2021.

The entries of the *Q* matrix are displayed in Table 3.

### 4.1. Application of the GAHP Method

For the calculation of the criteria weights, a group of four experts in the field is selected. Each of them will evaluate in pairs the criteria considered according to Saaty’s scale and build four comparisons in pairs matrices $G^{E_{1}},\;G^{E_{2}}$, $G^{E_{3}},\;G^{E_{4}}$ (Table 4).

Each matrix is verified for consistency based on Equation (7). All the matrices $G^{E_{1}},\;G^{E_{2}}$, $G^{E_{3}},\;G^{E_{4}}$ are consistent. The $CR^{E_{1}}=$ 0.086 for matrix $G^{E_{1}}$, $CR^{E_{2}}=$ 0.035 for matrix $G^{E_{2}}$, $CR^{E_{3}}=$ 0.098 for matrix $G^{E_{3}}$ and $CR^{E_{4}}=$ 0.085 for matrix $G^{E_{4}}$.

$G^{E_{1}},\;G^{E_{2}}$, $G^{E_{3}},\;G^{E_{4}}$ are aggregated with the geometric mean method (Equation (8)), and the aggregated comparison in pairs matrix are presented in Table 5.

The GAHP criteria weights for each expert and aggregated criteria weights are calculated using the Dominant Eigenvalue algorithm (Section 3.4). The results are presented in Table 6.

### 4.2. Application of the EEWM

**Figure 3:**
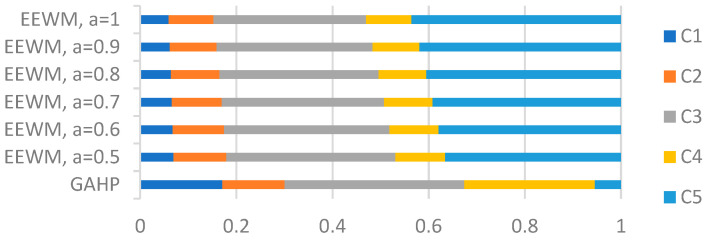
A comparison between the GAHP weights and EEWM weights.

The criteria weights calculated with EEWM (Equation (11) for different values of parameter a∈[0.5;1] are presented in Table 7. A comparison between the GAHP weights and EEWM weights for different values of *a* is displayed in Figure 3.

The objective nature of the EEWM shows a high value for the weight of criterion *C*_5_ compared to the weight of criterion *C*_5_ calculated with GAHP. The large value of the weight corresponding to criterion *C*_5_ calculated by EEWM is due to the fact that there is a great variability of data values for this criterion. For *a* = 1/2, the variability of the data values is smaller than that for *a* = 1 (Shannon entropy). The low value of the weight corresponding to *C*_5_ criterion calculated by GAHP is due to the subjectivity of the experts in evaluating the criteria. Experts considered that the criterion *C*_5_, new tests smoothed/1 million inhabitants, is less important than the other criteria.

### 4.3. Overall Criteria Weights Calculation

To make a compromise between the subjective involvement of experts and the objective evaluation, in the final calculation of weights, EEWM and GAHP are combined in Equation (12). The parameter *µ* controls the degree of subjectivity versus objectivity in calculating the weights of the criteria.

**Figure 4:**
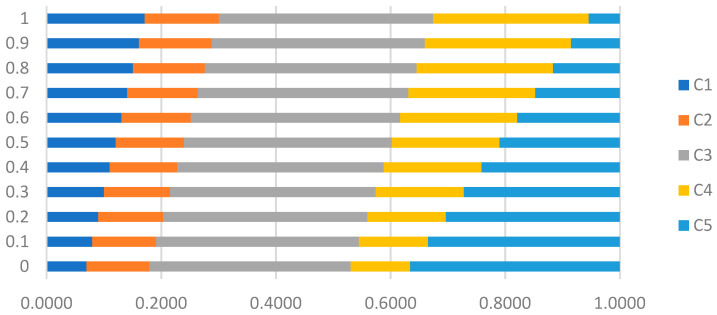
The criteria weights with variation of parameter *µ*.

The vector of overall criteria weights *W* is calculated with the EEWM and GAHP for different values of parameter *µ* and for *a* = 0.5 (Table 8). The differences of criteria weights for variations of parameter *µ* are presented in Figure 4.

The variation of the criteria weights depends on the importance of the objective evaluation of matrix Q (in EEWM) compared to the subjective evaluation of experts (in GAHP). When the parameter *µ* = 0, the criteria weights are calculated only with the EEWM. The objective character in the calculation of the criteria weights is maximum. Then, by varying the parameter *µ* from 0.1 to 0.9, the objective character gradually decreases, and the subjective character gradually increases. For *µ* = 1, the criteria weights are calculated only with the GAHP method. The subjective character in the calculation of the criteria weights is maximum.

The weights of criteria *C*_2_ and *C*_3_ vary less than weights of criteria *C*_1_, *C*_4_ and *C*_5_. The largest variation is for the *C*_5_ criterion.

### 4.4. Application of the COPRAS Method

For the fourth COVID-19 wave, the COPRAS solutions are calculated. These solutions are considered countries’ COVIND indicators.

For each value of parameter *µ*, a criteria weights set is considered (Table 8). For each criteria weights set, the COPRAS method is applied.

The vector *S* with COPRAS results for each criteria weights set is displayed in Table 9 (for the parameter *a* = 0.5).

The ranks of COPRAS results obtained with different criteria weights set are obtained based on Equations (13)–(17) (Table 10).

Countries from the group of Western European countries (*µ* = 0.8, *µ* = 0.9 and *µ* = 1) occupy the first positions, and countries from the group of Eastern Europe occupy the last positions. The same rank is observed, whatever the value of *µ*, for Serbia and France.

**Figure 5:**
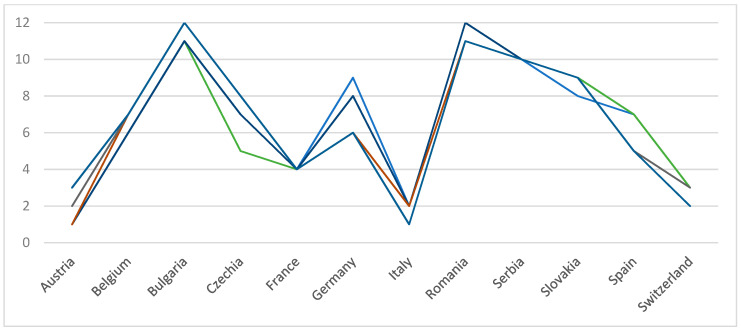
The variation of countries COVIND ranks for *a* = 0.5 and μ∈{0;0.1;0.2;0.3;0.4;0.5;0.6;0.7;0.8;0.9;1}.

As the degree of objectivity, in the calculation of the criteria weights, decreases, the countries’ ranks change. For *µ* = 0.1, *µ* = 0.2, *µ* = 0.3, *µ* = 0.4 and *µ* = 0.5, there are no changes in countries ranks. The greater differences are for *µ* = 0.8, *µ* = 0.9 and *µ* = 1 when the subjective character in the criteria calculation is high. It is observed that great variations are from Germany and Czechia (three positions) and Austria and Spain (two positions) (Figure 5).

### 4.5. Comparison of COPRAS Ranks Obtained with EWM and EEWM

A comparison of the COPRAS ranks obtained with EWM (*a* = 1) and EEWM for a∈{0.5;0.6;0.7;0.8;0.9} is presented in Table 11.

A difference of one position is for Bulgaria, Slovakia, Spain and Serbia.

The ranks of results obtained with VIKOR and TOPSIS multi-criteria methods for a∈{0.5;0.6;0.7;0.8;0.9;1} are presented in Table 12.

By comparing the COPRAS with VIKOR and TOPSIS, it is observed that the smallest difference in rank change is between COPRAS and TOPSIS for EEWM with *a* = 0.5.

### 4.6. A Comparative Analysis with General Countries’ Indicators

A comparative analysis of the COVIND indicator ranks with general countries’ indicators is realized based on Spearman correlations. The general countries indicators are: number of people fully vaccinated/per million (*I*_1_), stringency index (*I*_2_), population density (*I*_3_), median age (*I*_4_), aged 65 older (*I*_5_) and GDP per capita (Gross Domestic Product per capita (per person)) (*I*_6_).

The Stringency Index is a composite measure based on nine metrics. The metrics used are: school closures; workplace closures; cancellation of public events; restrictions on public gatherings; closures of public transport; stay-at-home requirements; public information campaigns; restrictions on internal movements; and international travel controls [71]. The index is calculated as the mean score of these metrics, each taking a value between 0 and 100. A higher score indicates a stricter response to the SARS-CoV 2 pandemic [71].

The values of these indicators and indicators ranks are presented in Table 13.

The COVIND ranks obtained with criteria weights set for μ∈{0;0.1;0.2;0.3;0.4;0.5;0.6;0.7;0.8;0.9;1} and *a* = 0.5 are compared with the ranks for general indicators. The Spearman correlations are calculated (Table 14).

In all cases, the highest correlation is for *I*_6_—GDP per capita and then for *I*_1_—number of people fully vaccinated/per million.

## 5. Conclusions

The paper proposes a multi-criteria decision support (MCDS) implemented to solve the problem of ranking a group of countries based on a new country COVID-19 complex indicator called COVIND in the COVID-19 fourth wave.

The experts are selected by a manager or decision maker. The parameter that controls the trade-off between the subjective and objective weighting helps decision makers face various situations in which the confidence in experts’ evaluations is essential. The value of parameter can be set by the decision maker or analyst depending on the degree of trust in the experts’ expertise.

Applying MCDS could lead to a better understanding of the factors involved in the COVID-19 pandemic on population health in a cumulative way. The information gained from this research can be used for support efforts and to justify public health spending. The decision makers can propose interventions and health policies based on scientific knowledge.

This COVIND indicator can better reflect the situation of a country in a group of countries or on a continental level in a desired period of time or wave. Based on COVIND results, decision makers can make more informed decisions and set strategies and policies to combat the pandemic.

MCDS can also be applied to other areas where it is necessary to rank the alternatives evaluated according to several criteria.

The contributions of the paper can be summarized as follows:

- A multi-criteria decision support (MCDS) based on EEWM, GAHP and COPRAS was proposed.
- An extended method called EEWM for computing the objective weights of the criteria was proposed. This method extends the classical EWM method by using an entropy function, which generalizes the classical Shannon entropy function. The EEWM is used in combination with GAHP and COPRAS in the MCDS.
- In order to emphasize the compromise between the subjectivism of experts and the objectivism of evaluation matrix, a parameter was introduced. The parameter can be varied in the range [0; 1] to achieve the compromise between the two methods for determining the criteria weights.
- The proposed MCDS is applied for the calculation of a complex indicator COVIND for a group of European countries in the fourth wave COVID-19.
- An analysis of the obtained MCDS rankings was realized:
- By the variation of the parameter that combines the EEWM and GAHP weights;
- By the variation of the parameter of extended entropy;
- By using alternative multi-criteria methods VIKOR and TOPSIS.

A Spearman correlation analysis with general country indicators such as the number of people fully vaccinated/per million, stringency index, population density, median age, aged 65 older and GDP (Gross Domestic Product) per capita per capita is realized.

By varying the two parameters (parameter a from the extended entropy function and parameter *µ* from the linear combination of weights), several ranking alternatives are obtained. They allow decision makers to analyze different evaluations of the fourth wave of COVID-19.

An interesting development of our study in the spirit of the papers [72] is the approach of consensus issues in the evaluators group and the consideration of the situations when experts show their self-confidence levels by referring to consensus reaching for group decision making with multi-granular unbalanced linguistic information. A minimum adjustment optimization model based on bounded confidence may be developed.

## Figures and Tables

**Table 1:** AHP Saaty scale [38,39].

Scale	Criteria
1	Equal Importance
3	Moderate Importance
5	Strong Importance
7	Very Strong Importance
9	Absolute Importance
2, 4, 6, 8	Intermediate values
1/2, …, 1/9	Reciprocals of above

**Table 2:** Average random consistency index [38,39].

Number of Criteria	3	4	5	6	7	8	9
Average RI	0.58	0.90	1.12	1.24	1.32	1.41	1.45

**Table 3:** The *Q* matrix for the fourth COVID wave.

European Countries	*C*_1_	*C*_2_	*C*_3_	*C*_4_	*C*_5_
Austria	308.4294	378.1481	1.8043	28.0773	40344.41
Belgium	334.3022	434.6661	1.3662	26.9196	5489.175
Bulgaria	212.2888	267.2547	10.6268	62.8098	3425.509
Czechia	235.7848	372.5565	2.5862	23.8699	8749.032
France	191.4035	176.2033	0.8478	22.6953	7687.89
Germany	157.3036	213.8961	1.2861	23.0129	1812.396
Italy	69.1924	68.6557	0.6042	6.1044	3821.611
Romania	193.1306	222.6808	7.53	46.6847	2116.905
Serbia	326.6165	466.465	4.3935	20.9965	2211.028
Slovakia	413.3236	603.5366	4.1888	20.776	6302.605
Spain	245.1909	229.3405	1.1545	25.1098	2722.29
Switzerland	156.6182	157.1383	0.3978	16.0821	3174.591

**Table 4:** Comparison in pairs matrices (a) $G^{E_{1}}$, (b) $G^{E_{2}}$, (c) $G^{E_{3}}$, (d) $G^{E_{4}}$.

**(a)**						**(b)**					
$G^{E_{1}}$	***C*** **_1_**	***C*** **_2_**	***C*** **_3_**	***C*** **_4_**	***C*** **_5_**	$G^{E_{2}}$	***C*** **_1_**	***C*** **_2_**	***C*** **_3_**	***C*** **_4_**	***C*** **_5_**
***C*** **_1_**	1	2	1	1	1	***C*** **_1_**	1	1	1/2	1/2	3
***C*** **_2_**	1/2	1	1	1	2	***C*** **_2_**	1	1	1/2	1/2	1
***C*** **_3_**	1	1	1	2	3	***C*** **_3_**	2	2	1	2	5
***C*** **_4_**	1	1	1/2	1	5	***C*** **_4_**	2	2	1/2	1	5
***C*** **_5_**	1	1/2	1/3	1/5	1	***C*** **_5_**	1/3	1	1/5	1/5	1
**(c)**						**(d)**					
$G^{E_{3}}$	***C*** **_1_**	***C*** **_2_**	***C*** **_3_**	***C*** **_4_**	***C*** **_5_**	$G^{E_{4}}$	***C*** **_1_**	***C*** **_2_**	***C*** **_3_**	***C*** **_4_**	***C*** **_5_**
***C*** **_1_**	1	3	1/3	1/3	7	***C*** **_1_**	1	1	1/3	1/5	9
***C*** **_2_**	1/3	1	1/5	1	3	***C*** **_2_**	1	1	1/5	1/5	5
***C*** **_3_**	3	5	1	2	6	***C*** *_3_*	3	5	1	2	9
***C*** **_4_**	3	1	1/2	1	5	***C*** **_4_**	5	5	1/2	1	7
***C*** **_5_**	1/7	1/3	1/6	1/5	1	***C*** **_5_**	1/9	1/5	1/9	1/7	1

**Table 5:** The aggregated comparison in pairs matrix.

Criteria	*C*_1_	*C*_2_	*C*_3_	*C*_4_	*C*_5_
***C*** **_1_**	1.000	1.565	0.485	0.427	3.708
***C*** **_2_**	0.639	1.000	0.376	0.562	2.340
***C*** **_3_**	2.060	2.659	1.000	2.000	5.335
***C*** **_4_**	2.340	1.778	0.500	1.000	5.439
***C*** **_5_**	0.270	0.427	0.187	0.184	1.000

**Table 6:** Experts’ criteria weights.

	*C*_1_	*C*_2_	*C*_3_	*C*_4_	*C*_5_
***W^E^*** **^1^**	0.2245	0.1830	0.2611	0.2302	0.1013
***W^E^*** **^2^**	0.1601	0.1336	0.3594	0.2724	0.0745
***W^E^*** **^3^**	0.1938	0.1128	0.4112	0.2414	0.0408
***W^E^*** **^4^**	0.1258	0.0941	0.4064	0.3446	0.0292
***W* ^(*GAHP*)^**	0.1712	0.1294	0.3736	0.2717	0.0541

**Table 7:** Criteria weights calculated by EEWM for different values of parameter *a*.

*a*	*C*_1_	*C*_2_	*C*_3_	*C*_4_	*C*_5_
0.5	0.0698	0.1094	0.3518	0.1035	0.3656
0.6	0.0680	0.1065	0.3440	0.1021	0.3794
0.7	0.0662	0.1038	0.3373	0.1006	0.3921
0.8	0.0642	0.1008	0.3309	0.0990	0.4051
0.9	0.0619	0.0975	0.3243	0.0970	0.4193
1	0.0592	0.0935	0.3170	0.0946	0.4357

**Table 8:** The overall criteria weights *W* for *a* = 0.5.

*µ*	*C*_1_	*C*_2_	*C*_3_	*C*_4_	*C*_5_
0	0.0698	0.1094	0.3518	0.1035	0.3656
0.1	0.0799	0.1114	0.3540	0.1203	0.3345
0.2	0.0901	0.1134	0.3561	0.1371	0.3033
0.3	0.1002	0.1154	0.3583	0.1539	0.2722
0.4	0.1103	0.1174	0.3605	0.1708	0.2410
0.5	0.1205	0.1194	0.3627	0.1876	0.2099
0.6	0.1306	0.1214	0.3649	0.2044	0.1787
0.7	0.1408	0.1234	0.3671	0.2212	0.1476
0.8	0.1509	0.1254	0.3692	0.2381	0.1164
0.9	0.1611	0.1274	0.3714	0.2549	0.0853
1	0.1712	0.1294	0.3736	0.2717	0.0541

**Table 9:** The COPRAS results for different criteria weights set.

European Countries	The COPRAS Results (with Different Criteria Weights Set) *a* = 0.5										
	*µ* = 0	*µ* = 0.1	*µ* = 0.2	*µ* = 0.3	*µ* = 0.4	*µ* = 0.5	*µ* = 0.6	*µ* = 0.7	*µ* = 0.8	*µ* = 0.9	*µ* = 1
Austria	1.000	1.000	1.000	1.000	1.000	1.000	1.000	1.000	0.876	0.712	0.551
Belgium	0.243	0.254	0.267	0.282	0.301	0.323	0.352	0.389	0.385	0.366	0.346
Bulgaria	0.099	0.101	0.103	0.106	0.110	0.114	0.119	0.126	0.118	0.105	0.093
Czechia	0.276	0.283	0.291	0.301	0.313	0.328	0.347	0.371	0.354	0.322	0.290
France	0.372	0.389	0.409	0.433	0.462	0.498	0.543	0.602	0.598	0.569	0.541
Germany	0.190	0.205	0.223	0.245	0.271	0.302	0.343	0.395	0.409	0.406	0.403
Italy	0.436	0.475	0.521	0.576	0.642	0.723	0.826	0.960	1.000	1.000	1.000
Romania	0.079	0.083	0.087	0.092	0.097	0.105	0.114	0.126	0.124	0.118	0.111
Serbia	0.101	0.106	0.113	0.121	0.131	0.143	0.158	0.178	0.180	0.174	0.168
Slovakia	0.192	0.197	0.202	0.208	0.216	0.226	0.238	0.254	0.241	0.218	0.196
Spain	0.215	0.229	0.246	0.267	0.292	0.322	0.361	0.411	0.420	0.412	0.404
Switzerland	0.413	0.442	0.475	0.515	0.564	0.625	0.702	0.802	0.823	0.810	0.798

**Table 10:** The ranks of COPRAS results obtained with different criteria weights set.

European Countries	The Rank of COPRAS Results (with Different Criteria Weights Set) *a* = 0.5										
	*µ* = 0 (EEWM)	*µ* = 0.1	*µ* = 0.2	*µ* = 0.3	*µ* = 0.4	*µ* = 0.5	*µ* = 0.6	*µ* = 0.7	*µ* = 0.8	*µ* = 0.9	*µ* = 1 (GAHP)
Austria	1	1	1	1	1	1	1	1	2	3	3
Belgium	6	6	6	6	6	6	6	7	7	7	7
Bulgaria	11	11	11	11	11	11	11	12	12	12	12
Czechia	5	5	5	5	5	5	7	8	8	8	8
France	4	4	4	4	4	4	4	4	4	4	4
Germany	9	8	8	8	8	8	8	6	6	6	6
Italy	2	2	2	2	2	2	2	2	1	1	1
Romania	12	12	12	12	12	12	12	11	11	11	11
Serbia	10	10	10	10	10	10	10	10	10	10	10
Slovakia	8	9	9	9	9	9	9	9	9	9	9
Spain	7	7	7	7	7	7	5	5	5	5	5
Switzerland	3	3	3	3	3	3	3	3	3	2	2

**Table 11:** The ranks of COPRAS results obtained with EWM and EEWM.

European Countries	EEWM					EWM
	*a* = 0.5	*a* = 0.6	*a* = 0.7	*a* = 0.8	*a* = 0.9	*a* = 1
Austria	1	1	1	1	1	1
Belgium	6	6	6	6	6	6
Bulgaria	11	10	10	10	10	10
Czechia	5	5	5	5	5	5
France	4	4	4	4	4	4
Germany	9	9	9	9	9	9
Italy	2	2	2	2	2	2
Romania	12	12	12	12	12	12
Serbia	10	11	11	11	11	11
Slovakia	8	8	8	8	8	7
Spain	7	7	7	7	7	8
Switzerland	3	3	3	3	3	3

**Table 12:** The ranks of VIKOR and TOPSIS results obtained with EWM and EEWM.

European Countries	VIKOR						TOPSIS					
	EEWM					EWM	EEWM					EWM
	0.5	0.6	0.7	0.8	0.9	1	0.5	0.6	0.7	0.8	0.9	1
Austria	1	1	1	1	1	1	1	1	1	1	1	1
Belgium	5	5	5	5	4	4	6	6	6	6	6	6
Bulgaria	12	12	12	12	12	12	12	12	12	12	12	12
Czechia	3	3	3	3	3	3	5	4	3	3	3	3
France	2	2	2	2	2	2	2	2	2	2	2	2
Germany	9	9	9	9	9	9	8	8	8	8	8	8
Italy	4	4	4	4	5	5	3	3	4	4	4	4
Romania	11	11	11	11	11	11	11	11	11	11	11	11
Serbia	10	10	10	10	10	10	10	10	10	10	10	10
Slovakia	8	7	7	7	7	7	9	9	9	9	9	9
Spain	7	8	8	8	8	8	7	7	7	7	7	7
Switzerland	6	6	6	6	6	6	4	5	5	5	5	5

**Table 13:** The values of general indicators for the group of European countries.

**European Countries**	***I*** **_1_**	***I*** **_1_** **Rank**	***I*** **_2_**	***I*** **_2_** **Rank**	***I*** **_3_**	***I*** **_3_** **Rank**	***I*_4_**	***I*** **_4_** **Rank**	***I*** **_5_**	***I*** **_5_** **Rank**	***I*** **_6_**	***I*** **_6_** **Rank**
Austria	59,171.006	4	56.457	4	106.749	8	44.4	5	19.202	6	45,436.686	2
Belgium	66,140.159	1	46.863	6	375.564	1	41.8	10	18.571	8	42,658.576	4
Bulgaria	20,621.523	12	41.222	10	65.18	12	44.7	4	20.801	3	18,563.307	11
Czechia	51,762.378	8	34.712	12	137.176	5	43.3	6	19.027	7	32,605.906	8
France	53,305.963	6	57.74	3	122.578	6	42	9	19.718	4	38,605.671	5
Germany	60,714.67	3	60.345	1	237.016	2	46.6	2	21.453	2	45,229.245	3
Italy	56,522.685	5	59.984	2	205.859	4	47.9	1	23.021	1	35,220.084	6
Romania	30,956.606	11	51.767	5	85.129	10	43	8	17.85	10	23,313.199	10
Serbia	41,966.813	10	37.51	11	80.291	11	41.2	11	17.366	11	14,048.881	12
Slovakia	42,070.169	9	42.319	9	113.128	7	41.2	11	15.07	12	30,155.152	9
Spain	62,134.074	2	46.704	7	93.105	9	45.5	3	19.436	5	34,272.36	7
Switzerland	52,368.273	7	44.393	8	214.243	3	43.1	7	18.436	9	57,410.166	1

**Table 14:** The Spearman correlations between COVIND ranks and the general indicators.

***µ***	***I*** **_1_**	***I*** **_2_**	***I*** **_3_**	***I*** **_4_**	***I*** **_5_**	***I*** **_6_**
0	0.5245	0.3007	0.5035	0.2312	0.2727	0.7203
0.1	0.5664	0.3566	0.5385	0.2977	0.3427	0.7622
0.2	0.5664	0.3566	0.5385	0.2977	0.3427	0.7622
0.3	0.5664	0.3566	0.5385	0.2977	0.3427	0.7622
0.4	0.5664	0.3566	0.5385	0.2977	0.3427	0.7622
0.5	0.5664	0.3566	0.5385	0.2977	0.3427	0.7622
0.6	0.6503	0.4266	0.4825	0.3398	0.3706	0.7762
0.7	0.6783	0.5734	0.5105	0.3958	0.3986	0.8252
0.8	0.6713	0.5874	0.5385	0.4238	0.4336	0.7972
0.9	0.6503	0.5594	0.5734	0.4098	0.4126	0.8042
1	0.6503	0.5594	0.5734	0.4098	0.4126	0.8042
